# Increased Density of Mobile Health Unit Encounters Among Primary Care Health Professional Shortage Areas

**DOI:** 10.3390/ijerph23040457

**Published:** 2026-04-03

**Authors:** Phillip D. Levy, Michael J. Twiner, Bethany Foster, Mallory Lund, Naitik Nilesh-Shah, Paul J. Kurian, Brian Reed, Anna Steinberg-Abreu, James L. Young, Robert D. Brook, Steven J. Korzeniewski

**Affiliations:** 1 Integrative Biosciences Center, Wayne State University, Detroit, MI 48202, USA; 2 Department of Emergency Medicine, Wayne State University, Detroit, MI 48202, USA; 3 Division of Cardiovascular Disease, Department of Internal Medicine, Wayne State University, Detroit, MI 48202, USA

**Keywords:** population health, health services research, ecological study, social vulnerability

## Abstract

**Highlights:**

**Public health relevance—How does this work relate to a public health issue?**

- Multiple projections signal increasing primary care provider shortages in the US over the coming decade.

**Public health significance—Why is this work of significance to public health?**

- Mobile health unit outreach to communities facing primary care health professional shortages might help to mitigate projected accessibility gaps.

**Public health implications—What are the key implications or messages for practitioners, policy makers and/or researchers in public health?**

- Our findings support the possibility that engagement with mobile health unit providers is increased in communities facing primary care health professional shortages.
- Additional efforts to scale and evaluate the capacity for mobile health units to help address projected primary care shortages appear warranted.

**Abstract:**

Mobile health units (MHUs) can reach populations facing barriers to traditional primary care, but information about factors associated with their utilization is limited. The objective of this ecological study was to evaluate whether MHU encounter density is increased in census tracts designated as Primary Care Health Professional Shortage Areas (HPSAs) and explore whether associations varied by socioeconomic vulnerability. We analyzed Wayne State University/Wayne Health MHU encounters with adult patients from July 2021 to September 2025. Negative binomial regression models with a log link and log(population) offset tested the a priori hypothesis that encounter density was increased in designated versus undesignated HPSA census tracts. Sensitivity analyses assessed variation by social vulnerability index score quartiles established by the US Centers for Disease Control and Prevention. One quarter of the five-county metropolitan Detroit, Michigan, catchment area census tracts were designated healthcare shortage areas. Overall, 13,852 encounters with 10,924 unique patients occurred across 924 of 1305 census tracts. Encounter rate per adult population was significantly increased by severalfold comparing designated versus undesignated shortage areas, with stronger associations at lower socioeconomic vulnerability index score quartiles (interaction *p* = 0.0006). These findings support continued efforts to scale and evaluate MHUs to address projected healthcare shortages, particularly in socioeconomically vulnerable areas.

## 1. Introduction

Mobile health units (MHUs) provide healthcare services in communities by deploying mobile clinics rather than relying on patients to present for care at traditional “brick and mortar” locations [1,2]. By bringing services to locations where people live and work, MHUs can help address structural barriers that limit engagement with traditional healthcare systems, including transportation constraints, limited provider availability, cost barriers, and competing social needs [3,4]. In turn, MHUs are often used by health systems, public health agencies, and community organizations to improve healthcare access specifically among vulnerable populations (e.g., unhoused individuals [5], people struggling with substance use disorders [6]) often in underserved areas (e.g., conflict zones [7], natural disaster areas [8], rural settings [9,10,11], and urban areas with increased social vulnerability [12]).

Despite growing interest in MHUs as a strategy to expand healthcare access, empirical evidence describing the geographic and socioeconomic factors associated with their utilization is limited. In particular, little is known about whether MHU services might help to address federally designated Primary Care Health Professional Shortage Areas (HPSAs). The United States introduced the HPSA program in 1965 with hopes of increasing physician access in areas designated as underserved [13], ultimately to address what became increasingly recognized geographic health disparities (e.g., [14]). Despite the program’s 50-plus-year history, optimal approaches for addressing HPSAs is an ongoing debate with recent studies showing little or no evidence of impact on physician density or associated health outcomes [15]. This prompted our curiosity about the potential for MHUs to help address healthcare provider shortages.

Our mobile health program targets vehicle deployment to areas with heightened social vulnerability in Southeastern Michigan, USA [12]. Here we examined whether MHU encounter density was increased in HPSA-designated census tracts within our catchment. Given our deployment strategy and prior evidence linking social vulnerability to healthcare access [16,17,18,19], we further assessed whether observed associations varied across areas with different levels of socioeconomic vulnerability.

Our descriptive study is guided by the premise that HPSA designation reflects structural limitations in healthcare access that may influence patterns of MHU utilization, potentially differing across communities with varying levels of socioeconomic vulnerability.

## 2. Materials and Methods

### 2.1. Study Design

The Institutional Review Board of Wayne State University approved this study, determining that it met criteria for a waiver of informed consent per applicable federal regulations (45 CFR 46.116).

Clinical data were provided by the Wayne State University/Wayne Health MHU program that is described in detail elsewhere [12,20,21]. Briefly, a fleet of five-to-seven MHUs are deployed five-to-six days per week to site locations that are prioritized using our interactive map web application (the PHOENIX [22] Prevalence Profiler [23]) based on high social vulnerability index scores [12] and high rates of hypertension, diabetes, and high cholesterol estimated by the Centers for Disease Control and Prevention [24]. After intake, a focused medical history is performed by staff including community health workers, nurses, and research assistants following standardized questionnaires. Patients can then opt to undergo blood pressure (BP) evaluation and/or laboratory testing for cardiometabolic disorder risk factors (e.g., non-fasting lipoprotein measurements and glycated hemoglobin (HbA1c) assessment). BP screening and venous blood draws are performed onsite and biological samples are shipped within 24 h to one of our partner clinical laboratories for analyses (https://www.questdiagnostics.com/; https://www.labcorp.com/ (accessed on 29 October 2025)). Clinical histories and BP measurements are entered into Wayne Health’s electronic medical record (EMR) system onsite, while laboratory results are automatically imported into the EMR approximately 1–3 days later.

This descriptive ecological study leverages deidentified data extracted from Wayne Health’s EMR for adult patient encounters that included BP evaluation or laboratory testing from July 2021 to September 2025. The focus on encounters with BP or laboratory measurements was intended to limit potential bias associated with pandemic era COVID-19 testing activities. We further restricted the analysis to encounters with patients living in Detroit, Michigan, USA, or surrounding counties (Wayne, Macomb, Oakland, Washtenaw, and Monroe).

### 2.2. Geocoding

Residential street addresses were geocoded to identify the census tract Federal Information Processing Standards (FIPS) code using a three-stage process. First, column names were standardized to the US Census Bureau requirements (Street, City, State, ZIP) and basic filtration was applied to remove empty/duplicate rows before processing. Second, batches of up to 6000 distinct addresses were sent directly to the US Census Bureau Batch Geocoder API. Third, unmatched addresses were screened for typos, formatting errors, or missing elements, and the corrected addresses were submitted to the US Census Bureau Batch Geocoder API to retrieve the 11-digit FIPS code for each street address. The residential street address was chosen because we hypothesized that MHU encounters would be increased among people living in neighborhoods designated as healthcare shortage areas.

### 2.3. Census Tract Characteristics

Primary Care Health Professional Shortage Area (pcHPSA) designation data were provided by HRSA. We used the most recent designation for each area based on the date the record was last updated as of 28 August 2025, when the data were downloaded. The pcHPSA-designated census tracts were compared to undesignated areas. We also classified census tracts by shortage severity using information from a Healthcare Professional Shortage Area score that ranges from 1 to 26 with higher scores indicating greater need. Undesignated census tracts were compared to those designated as mild shortage (bottom quartile HPSA score < 12), moderate shortage (middle quartile HPSA score 12- < 19) or severe shortage areas (top quartile HPSA score ≥ 19).

Based on prior knowledge of association with healthcare utilization, we also included information from an index of census tract vulnerability related to socioeconomic status (SES) [i.e., poverty, low income, unemployment, and below high school education] provided by the latest available CDC social vulnerability index (SVI) dataset from 2022 [25]. The SES SVI score was classified by quartile to represent low (Q1, <25th percentile), moderate (Q2 and Q3, 25th-to-<75th percentile) and high (Q4, ≥75th percentile) vulnerability. Additional information about adult population size was provided by the 2018–2022 American Community Survey 5-year estimate.

### 2.4. Statistical Analysis

This ecological study used census tracts as the unit of analysis. Individual MHU encounters were aggregated by patients’ home address census tract to examine geographic variation in service utilization. An ecological design was chosen because the main exposure—Primary Care Health Professional Shortage Area (HPSA) designation—is defined at the census tract level, and we aimed to evaluate tract-level variation in encounter density. Due to the absence of individual-level data from non-users of MHU services, we were unable to examine our research questions using a multilevel model.

First, we summarized the characteristics of patients presenting for care to provide context for the observed encounter counts. Frequencies and percentages were calculated for categorical data and medians and interquartile range (IQR) boundaries (i.e., 25th and 75th percentiles) were calculated for interval data. Next, we aggregated the total number of MHU encounters by patients’ home address census tract. An encounter was defined as an individual patient MHU visit on a given date of service, irrespective of the number or type of services rendered; multiple services delivered to an individual patient during the same visit were counted as a single encounter, and individuals were able to have multiple encounters on different visit dates. Magnitudes of association with MHU encounter density and 95% confidence intervals (CIs) were estimated using negative binomial regression with a log link to account for overdispersion observed in the data (Pearson χ^2^/df > 1 under a Poisson model) and a log(adult population) offset to estimate per capita rates. Because socioeconomic vulnerability is associated both with HPSA designation and MHU deployment, SES SVI category was included as an independent variable in an adjusted model that was specified as follows:

$$\log\left(E\left(Y_{i}\right)\right)=\beta_{0}+\sum_{k=1}^{K-1}\beta_{1k}{\mathrm{pcHPSA}}_{\mathrm{ik}}+\sum_{m=1}^{M-1}\beta_{2m}{\mathrm{SVI}}_{\mathrm{im}}+\log\left({\mathrm{Population}}_{i}\right)$$

We also conducted sensitivity analyses by adding an interaction term to test whether the magnitude of association varied by the SES SVI score quartile group as follows:

$$\log\left(E\left(Y_{i}\right)\right)=\beta_{0}+\sum_{k=1}^{K-1}\beta_{1k}{\mathrm{pcHPSA}}_{\mathrm{ik}}+\sum_{m=1}^{M-1}\beta_{2m}{\mathrm{SVI}}_{\mathrm{im}}+\sum_{k=1}^{K-1}\sum_{m=1}^{M-1}\beta_{3km}\left({\mathrm{pcHPSA}}_{\mathrm{ik}}\times {\mathrm{SVI}}_{\mathrm{im}}\right)+\log\left({\mathrm{Population}}_{i}\right)$$

For the model without interaction, exponentiated coefficients were interpreted as incidence rate ratios comparing each pcHPSA category and SES SVI category with their respective reference categories, holding other model terms constant. For the interaction model, the association between pcHPSA category and encounter rates was allowed to vary across SES SVI categories; therefore, stratum-specific incidence rate ratios were obtained from linear combinations of the relevant main-effect and interaction coefficients. Additional model justifications are provided in Supplemental Materials; assumptions were evaluated using standard diagnostics, including assessment of overdispersion (Pearson χ^2^/df), comparison of model fit across Poisson, negative binomial, and zero-inflated negative binomial models using AIC and BIC (Table S1), and inspection of residuals (Table S2). Multicollinearity among predictors was assessed using variance inflation factors and condition indices derived from the model design matrix, with standard thresholds used to identify potential concerns. Analyses were performed using SAS v9.4 (Raleigh, NC, USA), and statistical significance was defined using two-sided tests with α = 0.05.

## 3. Results

**Figure 1:**
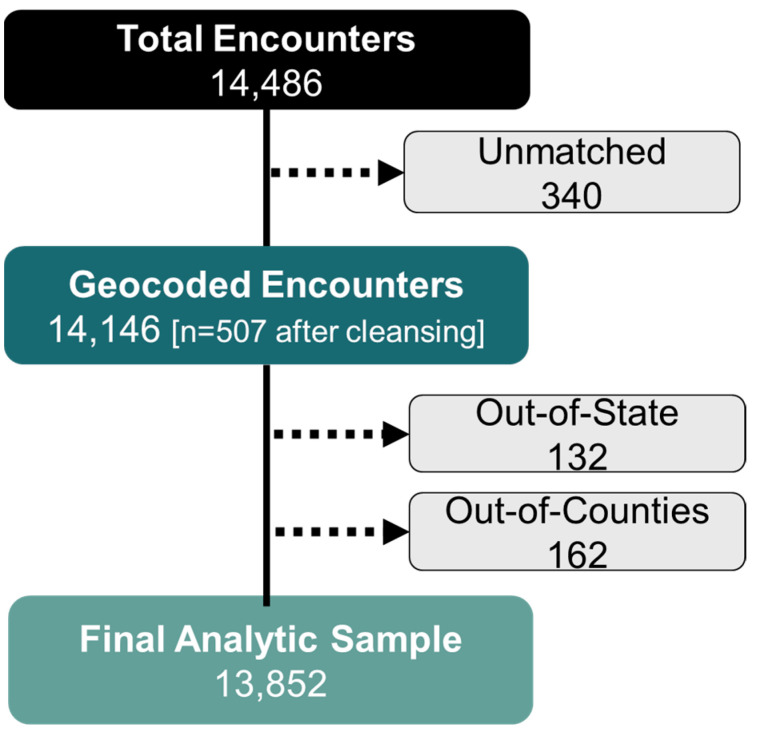
Geocoding flow chart with arrows indicating exclusions.

The home address was successfully geocoded to census tract FIPS codes for 98% of the 14,486 MHU encounters with BP or laboratory measurements during the study period [Figure 1]; *n* = 507 (3.6%) addresses required cleansing to correct misspellings (e.g., “Gratiot Ave” recorded as “Gratiot Avnue”), inconsistent abbreviations (e.g., “Street” vs. “St” vs. “Str”), concatenation (e.g., “123MainSt” instead of “123 Main St”), and inconsistent city naming (e.g., “Detroit” vs. “Detriot” or “City of Detroit”). Address cleansing was not associated with pcHPSA category (*p* = 0.76), socioeconomic status quartile group (*p* = 0.60), Black race (*p* = 0.96), age quartile group (*p* = 0.78), or biological sex (*p* = 0.62).

Geocoding failure occurred less frequently among encounters with patients who identified as Black versus non-Black (2% vs. 3%, *p* = 0.0002) but was not associated with biological sex (*p* = 0.36) or age quartile group (*p* = 0.18).

After excluding information from out-of-state (*n* = 132) and out-of-county (*n* = 162) residents, information from 13,852 encounters with 10,924 unique patients living in 924 unique census tracts was included. The majority of patients identified as Black or African American (71%), 61% were female and the median (IQR) age at first encounter was 57 (41–67) years. Most patients had a single encounter (85%), 10% had one repeat visit, and 5% had ≥3 encounters.

**Figure 2:**
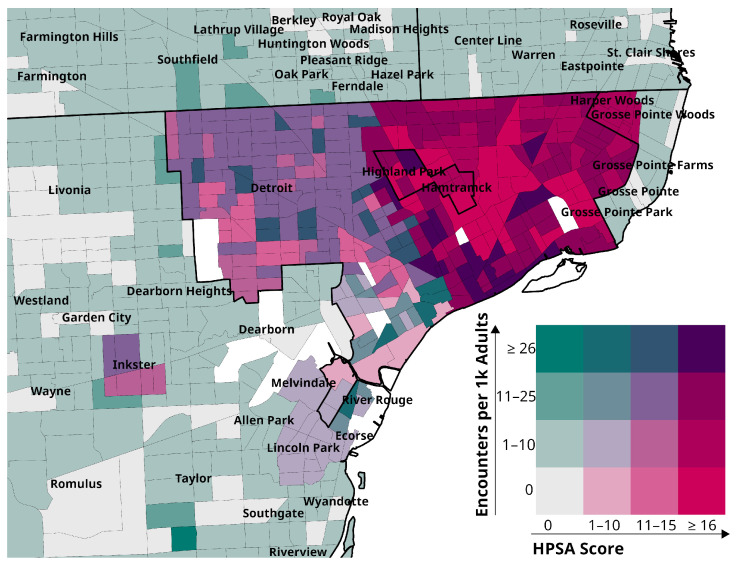
Density of mobile health unit encounters per 1 K adult population by Primary Care Health Professional Shortage Area (HPSA) score.

There were no encounters with patients who lived in 29% of the 1305 catchment area census tracts; among the remaining 924 tracts, the median (IQR) number was six (2–18) and 5% had ≥48 encounters. Approximately one quarter of the census tracts were designated pcHPSA (*n* = 336, 26%). Figure 2 maps the density of MHU encounters by pcHPSA score category.

The MHU encounter rate per adult population was significantly increased by approximately ninefold comparing pcHPSA-designated versus -undesignated census tracts (Table 1), indicating greater MHU service utilization. Adjustment for vulnerability related to socioeconomic status reduced the estimated magnitude by 25%, but the association remained statistically significant. Subclassification of the pcHPSA designation revealed no appreciable differences comparing mild, moderate and severe shortage areas versus undesignated census tracts.

Sensitivity analyses showed that the estimated magnitude of association between pcHPSA designation and the density of MHU encounters varied by SES SVI category (interaction *p*-value, 0.0006). Shortage areas were significantly associated with increased MHU encounter density in areas with low, middle or top SES SVI quartiles (rate ratio [95% CI]: 18 [6.4–52], 8 [6.3–11], and 4 [3–5.9], respectively). However, the estimated magnitude of association weakened with increasing socioeconomic vulnerability.

## 4. Discussion

Our group uses information from the CDC social vulnerability index and PLACES datasets to guide mobile health deployment to areas in need of services including blood pressure testing and lipid panel evaluation [12]. We conducted this ecological study to test for differences in the number of visits among communities facing specific healthcare shortages. Our main finding is that the density of mobile health unit encounters was increased by severalfold in our sample of census tracts among Primary Care Health Professional Shortage Areas designated by the US Health Resources and Services Administration. To our knowledge this is the first report of an association between primary healthcare provider shortage areas and an increased density of MHU encounters.

We were unable to identify prior ecological studies leveraging formal geographic designations of healthcare shortage areas to investigate factors associated with mobile healthcare utilization. At least one person-level cohort study associated repeat mobile medical clinic visits with chronic illnesses and comorbidities but found no association with health insurance status after adjusting for demographic factors and medical history [26].

Key to the interpretation of our findings is that our MHU deployment was not random but guided by community-level indicators of need including social vulnerability indicators. In turn, the observed associations between primary care shortage designation, MHU encounter density and socioeconomic vulnerability likely reflect a combination of underlying need and intentional placement of services. Intentional MHU program targeting may amplify observed differences across communities, complicating any potential causal inferences. Accordingly, our results should be interpreted cautiously as descriptive of service utilization patterns within a strategically deployed system, rather than signaling truly independent effects of community characteristics on MHU encounter rates. Further, given our study design inherently risks ecological fallacy, no firm conclusions can be drawn about whether individual residents in shortage areas are more likely to use MHU services. The observed pattern of associations nevertheless prompts us to infer that additional research is warranted to investigate that possibility. Indeed, HRSA and the Association of American Medical Colleges (AAMC) project national shortages of 20,000-to-70,000 primary care providers in the US over the coming decade [27]. Moreover, evidence from previous studies is consistent with positive returns on investment (ROI) in mobile healthcare [4,28,29]. However, most MHU ROI estimates are derived from small non-randomized studies relying heavily on extrapolation from intermediate clinical markers and modeled counterfactuals that are difficult to empirically validate.

It is important to recognize that mobile healthcare programs are typically designed to complement traditional primary care, not replace it. Patients have reported that MHUs help them navigate complexities of the larger healthcare system and connect with local medical and social services [30,31]. However, key challenges limiting further scaling of mobile health program implementation includes financial instability, unique physical infrastructure costs (e.g., vehicle and ongoing maintenance fees), limitations related to environmental conditions (e.g., reduced encounters in poor weather), and care fragmentation without broader health system integration and data sharing infrastructure [1].

Designated primary care shortage areas in our sample were associated with increased MHU encounter density across communities with low, moderate, and high socioeconomic vulnerability; however, the magnitude of association was stronger in lower vulnerability areas. If independently replicated in general population samples, these findings may signal a need for additional strategies to optimize reach in high-vulnerability primary care shortage areas. Our group leverages community health workers and partners with community-based organizations [20], but the optimal approach for optimizing reach is a furtive area of ongoing investigations.

We observed no clear gradient in the association with MHU encounter density across shortage severity categories. We do not know why. The absence of a shortage severity gradient may indicate that binary designation status captures most relevant variation in access-related need. Equally plausible is that our targeted MHU deployment compressed differences across shortage area severity strata by increasing service availability in higher-need areas.

### Strengths and Limitations

A key strength of this study is the incorporation of formally designated primary care shortage area data, rather than reliance on anecdotal evidence or proxy measures to identify medically underserved areas. Despite a rather large sample size of more than ten thousand mobile health patients, our study is nevertheless limited by a small number of encounters per census tract. Consequently, the estimated magnitudes of associations may be inflated. It is also possible that some patients visited other mobile health clinics; however, we are unaware of other programs offering blood pressure and metabolic testing in the catchment area. Geocoding was unsuccessful for 2% (*n* = 340) of MHU encounters and occurred less frequently among patients who identified as Black versus non-Black (2% vs. 3%); although the absolute risk and race-difference were small, we cannot rule out the potential for bias. Additional limitations are that we were unable to include information about linkages to primary care or mobile health site characteristics, but both topics will be addressed in planned future studies, nor can we rule out the possibility of ecological bias (i.e., associations observed on an aggregate level may not represent or reflect associations on an individual level [32]). Further, like all observational studies, ours is unable to distinguish between associations and causation.

## 5. Conclusions

Mobile health unit encounters in our sample of metropolitan Detroit, Michigan, USA, census tracts were increased in designated versus undesignated Primary Care Health Professional Shortage Areas. The observed associations between primary care shortage designation, MHU encounter density and socioeconomic vulnerability likely reflects a combination of underlying need and data directed deployment of MHU services, rather than independent effects of shortage designation on encounter density. Further research is needed to determine the relative contributions of unmet need, deployment strategy, and other contextual factors.

## Figures and Tables

**Table 1:** Magnitudes of association between Primary Care Health Professional Shortage Areas and mobile health unit encounters.

Census Tract Classifications	Unadjusted			Adjusted		
	RR	95% LCL	UCL	RR	95% LCL	UCL
Binary HPSA Classification						
Designated Primary Care Health Professional Shortage Area	9.4	7.9	11.2	6.6	5.4	8.0
Undesignated (reference)	1			1		
						
Social vulnerability related to socioeconomic status (SES SVI)						
Top SES SVI quartile (Q4, ≥75th percentile)	-	-	-	2.3	1.9	2.8
Middle SES SVI quartiles (Q2 and Q3, 25th-to-<75th percentile)	-	-	-	2.8	2.3	3.6
Bottom SES SVI quartile (Q1, <25th percentile; reference)				1		
						
Categorical HPSA Classification						
Mild shortage (bottom quartile HPSA score < 12)	7.6	5.0	11.5	5.0	3.3	7.6
Moderate shortage (middle quartile HPSA score 12- < 19)	9.6	7.5	12.3	6.9	5.4	9.0
Severe shortage (top quartile HPSA score ≥ 19)	9.8	7.7	12.4	6.7	5.2	8.6
Undesignated (reference)	1			1		
						
Social vulnerability related to socioeconomic status (SES SVI)						
Top SES SVI quartile (Q4, ≥75th percentile)	-	-	-	2.3	1.9	2.8
Middle SES SVI quartiles (Q2 and Q3, 25th-to-<75th percentile)	-	-	-	2.9	2.3	3.6
Bottom SES SVI quartile (Q1, <25th percentile; reference)				1		

Note: HPSA, Health Professional Shortage Area; RR, rate ratio; LCL, lower 95% confidence limit; UCL, upper 95% confidence limit; SVI, social vulnerability index.

## Data Availability

The data presented in this study are available upon reasonable request to the corresponding authors and are not publicly available due to privacy protections.

## Supplementary Materials

The following supporting information can be downloaded at https://www.mdpi.com/article/10.3390/ijerph23040457/s1, Table S1: Criteria for Assessing Goodness of Fit; Table S2: Negative Binomial Regression Model Diagnostics.

## Abbreviations

The following abbreviations are used in this manuscript:

MHUs	Mobile Health Units
HRSA	U.S. Health Resources and Services Administration
BP	Blood Pressure
HbA1c	Non-Fasting Lipoprotein Measurements and Glycated Hemoglobin
EMR	Electronic Health Record
FIPS	Federal Information Processing Standards
API	Application Programming Interface
pcHPSA	Primary Care Health Professional Shortage Area
SES	Socioeconomic Status
SVI	Social Vulnerability Index
IQR	Interquartile Range
CIs	Confidence Intervals
AAMC	Association of American Medical Colleges
