# Supplementary material for: Increased Density of Mobile Health Unit Encounters Among Primary Care Health Professional Shortage Areas
Source: Int J Environ Res Public Health. 2026 Apr 3;23(4):457. doi: 10.3390/ijerph23040457 (PMC13116873; doi:10.3390/ijerph23040457)
Supplement: Supplementary file 1 [file ijerph-23-00457-s001.zip › ijerph-4194210-supplementary.pdf]

SUPPLEMENT MATERIALS

Table S1 shows that the Poisson model demonstrated substantial overdispersion (Pearson χ^2/df > 14) and poor fit (AIC > 18,000). In contrast, the negative binomial model appropriately accounted for dispersion (Pearson χ^2/df ≈ 1.1) and provided substantially improved fit. Zero-inflated negative models did not meaningfully impact model fit (ΔAIC < 5). Residual diagnostics for the negative binomial model (Supplementary Table S2) demonstrated adequate fit, with Pearson and deviance residuals centered near zero and exhibiting approximately unit variance. Multicollinearity was assessed using variance inflation factors (VIF) and condition indices, with no evidence of problematic collinearity (all VIF values < 2 and condition indices < 10).

Table S1. Criteria For Assessing Goodness Of Fit

MODEL SPECIFICAION Fit Criteria	Negative Binomial	Zero-Inflated Negative Binomial	Poisson
**UNADJUSTED**			
**AIC**	7464	7468	18155
**BIC**	7479	--	18165
**Pearson X^2/df**	1.13	--	15.81
**ADJUSTED**			
**AIC**	7374	7378	17507
**BIC**	7400	--	17528
**Pearson X^2/df**	1.13	--	14.43
**INTERACTION**			
**AIC**	7363	7367	17183
**BIC**	7399	--	17214
**Pearson X^2/df**	1.15	--	14.28

Note: AIC, Akaike information criterion; BIC, Bayesian information criterion; df, degrees of freedom; X^2, Chi-square.

Table S2. Negative Binomial Regression Model Diagnostics

MODEL SPECIFICAION	Residual	Mean	Standard Deviation	Minimum	Maximum
**UNADJUSTED**	Raw	-0.91	13.9	-55.93	128.78
	Pearson	0	1.07	-0.73	11.42
	Deviance	-0.43	0.96	-2.24	3.71
**ADJUSTED**	Raw	-1.06	14.2	-63.39	125.07
	Pearson	0	1.06	-0.77	10.53
	Deviance	-0.41	0.97	-2.35	3.6
**INTERACTION**	Raw	-0.88	13.76	-53.43	130.01
	Pearson	0	1.07	-0.77	10.87
	Deviance	-0.41	0.97	-2.33	3.68
